# American Medical Biography

**Published:** 1860-09

**Authors:** 


AMERICAN MEDICAL BIOGRAPHY.—Lindsay and Blakiston will publish, early in October, a large and beautiful volume of memoirs of eminent American physicians and surgeons, under the editorial management of Professor Gross.

